# Effects of variable bolt position of femoral neck system on Pauwels III fractures with superior femoral neck bone defect: multimodal biomechanical and clinical study

**DOI:** 10.3389/fsurg.2026.1914575

**Published:** 2026-10-21

**Authors:** Qing Zhang, Xu Zhang, Jiawen Wang, Liudong Zhang, Xiangyu Qi, Yongxiang Lv, Wenbo Li, Yazhong Zhang, Junqiang Wang, Zhian Liu, Ziqiang Zhu

**Affiliations:** 1 Department of Orthopaedics, The Second Affiliated Hospital of XuZhou Medical University, Xuzhou, China; 2 Graduate School of Xuzhou Medical University, Xuzhou, Jiangsu, China; 3 Department of Orthopaedics, Beijing Jishuitan Hospital, Beijing, China; 4 Department of Anatomy, School of Basic Medical Sciences, Xuzhou Medical University, Xuzhou, Jiangsu, China

**Keywords:** bone defect, finite element analysis, mechanical testing, Pauwels type III femoral neck fracture, surgical robot.

## Abstract

**Introduction:**

There is currently a lack of biomechanical evidence regarding whether the central position recommended by guidelines outperforms eccentric positions when using the Femoral Neck System (FNS) for Pauwels type III femoral neck fractures with superior neck defects. This study evaluated the biomechanical stability of FNS placement at different positions (anterior, posterior, central, and inferior) to guide clinical practice.

**Methods:**

A multimodal study combining finite element analysis (FEA) and cadaveric biomechanical testing was conducted on Pauwels III fracture models with 30° wedge-shaped defects in the superior neck. Furthermore, a retrospective analysis was performed on 27 patients who underwent robot-assisted precise central FNS fixation between 2021 and 2025, evaluating postoperative Garden alignment index and the 12-month Harris Hip Score (HHS).

**Results:**

FEA and mechanical testing revealed that eccentric FNS placements caused pronounced stress concentration and structural instability. Conversely, central FNS placement provided maximum structural stiffness and ideal stress distribution. Clinical application of this defect-oriented central strategy yielded excellent Garden alignment, 100% fracture union at the 12-month follow-up, and a mean HHS of 88.41.

**Discussion:**

In conclusion, for Pauwels type III fractures with superior femoral neck bone defects, precisely placing the bolt in the center of the femoral neck using an orthopedic surgical robot optimizes biomechanical properties and significantly improves fixation stability.

## 1 Introduction

Femoral neck fractures constitute approximately 50% of all hip fractures (1, 2). Particularly, increased attentions have been drawn to Pauwels type III fractures induced by high-energy injury in young and middle-aged adults (3–5). These fractures are frequently accompanied by bone defects that compromise the structural stability of the femoral neck and significantly increase the risk of internal fixation failure (6, 7). Bone defects in Pauwels type III fractures is morphologically heterogeneous rather than being confined to a single region of the femoral neck. In a CT-based morphological study, Jin et al. reported that among 84 Pauwels type III fractures, cortical comminution involved the posterior, inferior, superior, and anterior cortices in 77.4%, 65.5%, 41.7%, and 32.1% of cases, respectively (3). Therefore, although posterior and inferior defects predominate, superior cortical involvement remains a clinically relevant pattern, occurring in approximately two-fifths of Pauwels type III fractures. Similarly, quantitative virtual-reduction analysis of displaced femoral neck fractures demonstrated that bone defects may involve multiple regions, with the posterosuperior quadrant representing the most frequent main defect location (6). In Pauwels type III fractures, loss of superior cortical support may be particularly important because substantial vertical shear forces and bending moments must be transmitted across the fracture and fixation construct. Even after satisfactory reduction, a superior bone defect may prevent complete restoration of cortical contact, potentially increasing the mechanical dependence of the construct on implant position.

The FNS has emerged as an effective treatment modality for Pauwels type III fractures, owing to its superior resistance to shearing forces and rotational stability (8–10). Although current FNS application guidelines recommend placing the FNS centrally within the femoral neck, they fail to account for how bone defects affect the optimal spatial positioning of the FNS and, potentially, the overall postoperative stability (7, 11).

In order to address this clinical issue, We normalized the complex factors into a biomechanical system, combined with theoretical and practical stress analysis. Specifically, Pauwels type III fracture models was first constructed, featuring 30° wedge-shaped defects in the superior region of the femoral neck. After integrating FEA results with physical biomechanical testing, we systematically evaluated the impact of bone defects on the constructed models. Taken this into account, this strategy finally figures out optimal FNS placement for the superior defects, which is supposed to better guide the clinical fixation. Separately, the biomechanical concept was clinically corroborated by a retrospective evaluation of robot-navigated cases, in which robot navigation was employed to ensure precise FNS placement in a clinical setting (12–14). By providing high-precision FNS placement guidance, the robotic system facilitates superior execution of the preoperative FNS trajectory, and reduces the number of guide wire adjustments, which is supposed to avoid the risk of bone strength and insufficient FNS holding force (14, 15). In addition, this approach could shorten the overall operative time and also simultaneously minimize radiation exposure time (16, 17).

In summary, based on FEA and biomechanical testing, this study systematically evaluated the biomechanical applicability of central bolt placement in the presence of superior femoral neck defects, and investigated the effect of the spatial position of the FNS on the failure mode of internal fixation, thereby providing a theoretical basis for the clinical application of FNS in treating such fractures.

## 2 Materials and methods

### 2.1 Finite element model construction and validation

FEA model was constructed based on CT data obtained from a healthy adult. Image segmentation and three-dimensional (3D) reconstruction were performed using Mimics 21.0 (Materialise, Belgium) and Geomagic Studio 2021 (Geomagic, USA). The femoral model was modeled as a composite structure consisting of an internal cancellous core. Material property parameters are listed in Table 1 (18). The FNS was assigned titanium alloy material properties in accordance with the manufacturer's specifications (DePuy Synthes). To validate the model, the stiffness (746.30 N/mm) and peak stress distribution of the intact femur were compared with established literature data, showing excellent agreement (19, 20).

**Table 1:** Elastic modulus and Poisson's ratio of bone and internal fixation devices.

Variable	Elastic modulus (MPa)	Poisson's ratio
cortical bone	16800	0.3
cancellous bone	840	0.2
FNS	105000	0.35

### 2.2 Establishment of fracture and bone defect models

A Pauwels III fracture model featuring Pauwels angle of 60° was constructed (21), along with a 30° wedge-shaped bone defect adjacent to the fracture line in the upper femoral neck (Figures 1A,B) (6). The FNS was assembled in the femoral model (Figure 1C), with interfaces defined as frictional contacts (friction coefficients: bone-bone 0.46, bone-implant 0.42, implant-implant 0.20) (22, 23). Given the prohibitively high computational requirements imposed by a 1 mm mesh size on the analysis workstation, a mesh convergence study was conducted. The experimental results indicated minimal discretization error at a 1.4 mm element size, which was consequently selected as the optimal mesh configuration (Table 2).

**Figure 1:**
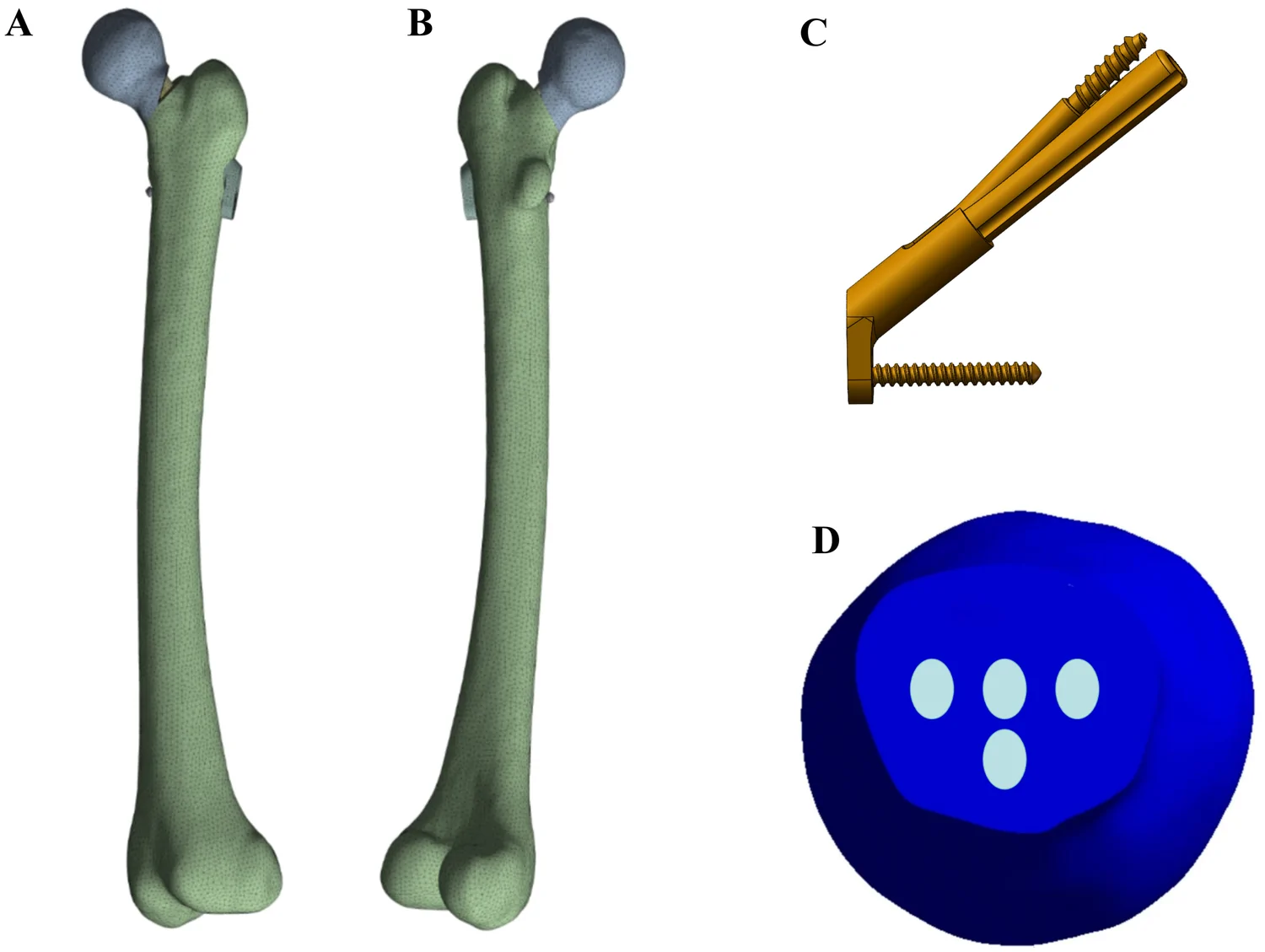
Schematic diagram of the fracture model. **(A,B)** fracture model; **(C)** Femoral Neck System (FNS); **(D)** Schematic illustration of bolt placement.

**Table 2:** Finite element mesh convergence size information.

Mesh size (mm)	Displacement (mm)	VMS (MPa)	Error (%)	Nodes	Elements
1	2.8121	22.319	0	5296704	3761564
1.2	2.812	22.662	1.54	3110953	2185177
1.4	2.8116	22.343	0.11	1990104	1382954
1.6	2.8114	22.092	−1.02	1365135	938934
1.8	2.8114	22.408	0.40	968494	658452
2.0	2.8109	22.515	0.88	729975	491531
2.5	2.8101	21.298	−4.57	388243	254354
3.0	2.8099	21.191	−5.05	240250	154013
4	2.8082	20.824	−6.70	113034	68852

### 2.3 Finite element analysis

A vertical load of 2100 N (three times body weight) was applied to the apex of the femoral head to simulate the single-leg stance phase, with the distal femur fully constrained (24, 25). This study evaluated the biomechanical stability of the FNS located in varying orientations (anterior, central, posterior, and inferior) in the presence of superior bone defects (Figure 1D). Primary outcomes included total displacement, Von Mises Stress (VMS), and relative inter-fragmentary displacement.

### 2.4 Cadaveric biomechanical testing

This study was approved by the Hospital Ethics Committee and conducted in full compliance with the ethical standards outlined in the 1964 Declaration of Helsinki and its subsequent amendments. Formalin-fixed cadaveric femoral specimens were screened to exclude prior pathology. Standardized osteotomies were performed on the bilateral femora of a single cadaveric donor to reproduce Pauwels type III femoral neck fractures with a 30° wedge-shaped superior bone defect, corresponding to the fracture and defect configuration established in the FEA model. With the assistance of a C-arm, the FNS was positioned either centrally or inferiorly within the femoral neck (Figure 2). Specimens were mounted in a physiological position on a servo-hydraulic testing machine (MTS Systems, USA) and subjected to axial compression at a rate of 2 mm/min until failure. Stiffness was calculated from the linear portion of the load-displacement curve. Failure was defined as the occurrence of fracture or a sudden drop in load.

**Figure 2:**
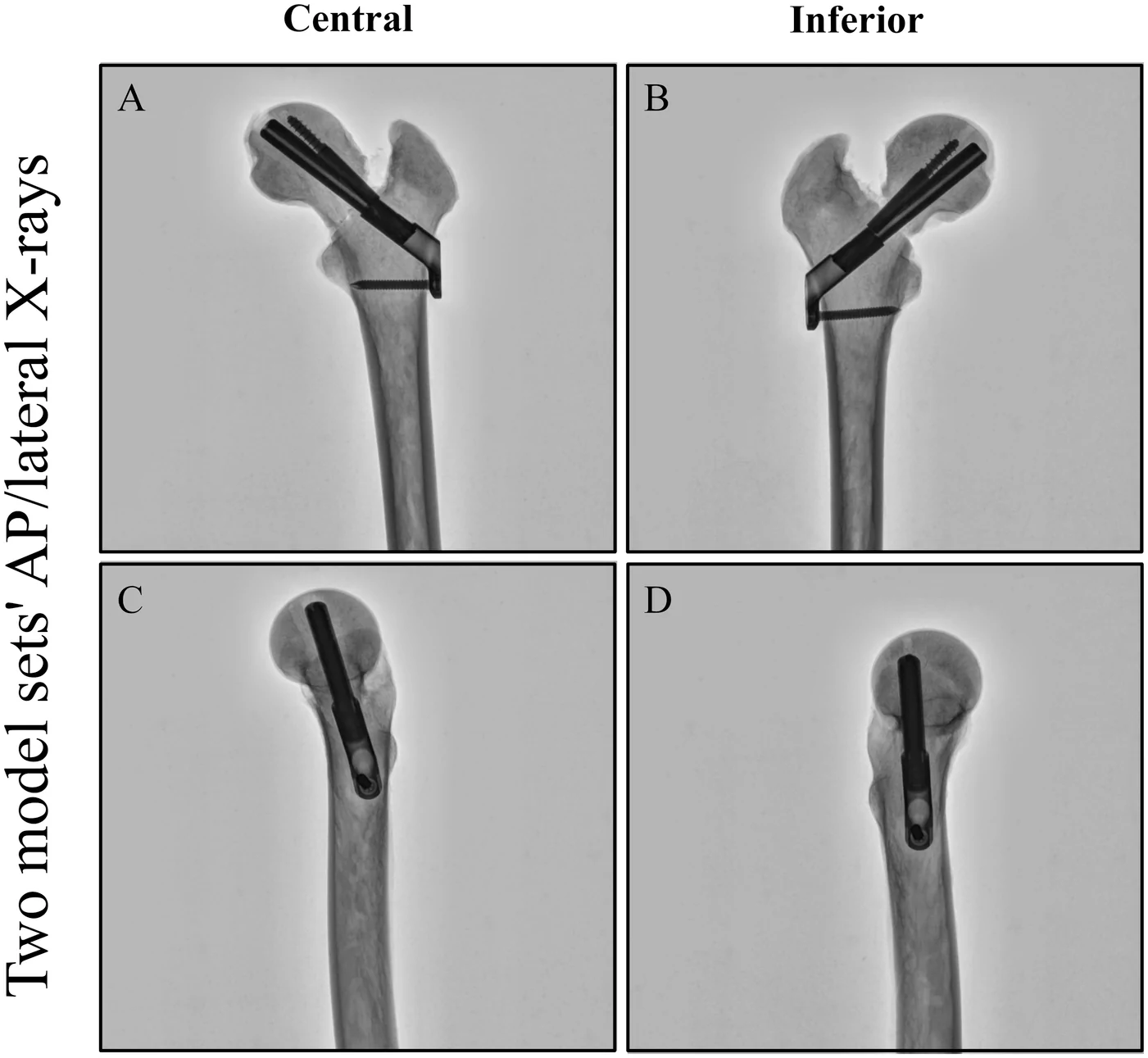
Anteroposterior and lateral x-ray radiographs of the central and inferior biomechanical models. **(A,C)** Anteroposterior and lateral views of the central model. **(B,D)** Anteroposterior and lateral views of the inferior model.

### 2.5 Clinical study design and surgical technique

This retrospective study evaluated 27 patients (aged 18−60 years) with Pauwels III femoral neck fractures and concurrent superior neck defects who underwent robot-guided femoral neck system (FNS) internal fixation between May 2021 and May 2025. The study received ethical approval. Based on preoperative radiographic assessment of the bone defect, the surgical trajectory for the FNS channel was planned along the central axis of the femoral neck. All surgical procedures were planned and executed using the TiRobot orthopedic surgical robot (TINAVI Medical Technologies Co., Ltd., Beijing, China). Intraoperatively, the standard FNS was implanted following guide wire insertion, which was directed by 3D fluoroscopic images registered to the robotic system. Postoperative radiographs were obtained to confirm reduction quality and implant positioning.

### 2.6 Postoperative management and assessment

Standard protocols for antibiotic prophylaxis and deep vein thrombosis (DVT) prevention were implemented postoperatively. The follow-up time points were 3, 6, and 12 months after surgery. Partial weight-bearing was permitted initially, while full weight-bearing could only be authorized after radiographic confirmation of fracture union (26–28). Clinical function was assessed using the Harris Hip Score (HHS) at 12 months follow-up. Fracture reduction quality was evaluated using the Garden Index.

### 2.7 Statistical analysis

Descriptive statistics were performed using Microsoft Excel (Microsoft, USA). Continuous variables are expressed as mean ± standard deviation.

## 3 Results

### 3.1 Finite element analysis results

#### 3.1.1 Total deformation and structural stiffness

Under a 2,100 N load, the total deformation of the central positioning model was 5.87 mm. In comparison, anterior, posterior and inferior positioning increased the deformation by 8.0%, 8.5% and 6.5%, respectively (Figure 3, Table 3).

**Figure 3:**
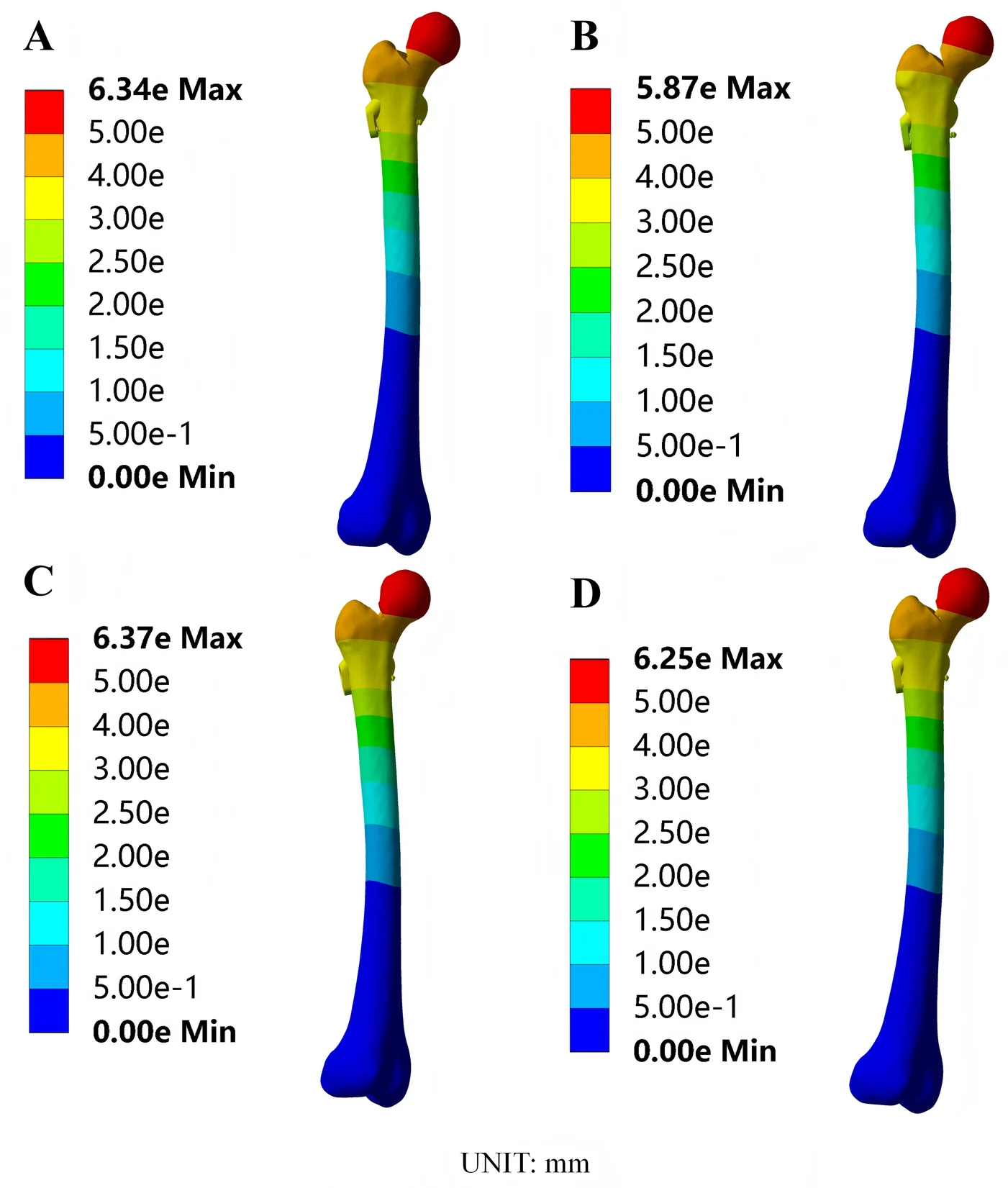
Total deformation diagram of the model with superior bone defect: **(A)** anterior bolt placement; **(B)** central bolt placement; **(C)** posterior bolt placement; **(D)** inferior bolt placement.

**Table 3:** Finite element analysis data.

Position	FNS VMS (MPa)	Femoral Head VMS (MPa)	Total Deformation (mm)	Displacement of Fracture Site (mm)
Anterior	404	52.9	6.34	0.533
Central	270	42.0	5.87	0.289
Posterior	391	52.0	6.37	0.429
Inferior	281	46.5	6.25	0.300

#### 3.1.2 Stress distribution on FNS

The peak VMS on the FNS for the central positioning was 270 MPa. Deviation from the central position led to a significant rise in stress, with increases of 43.8%, 39.1%, and 3.9% for anterior, posterior, and inferior positioning, respectively (Figure 4, Table 3). A lower stress distribution helps reduce the risk of fatigue fracture of the internal fixation device (22, 29).

**Figure 4:**
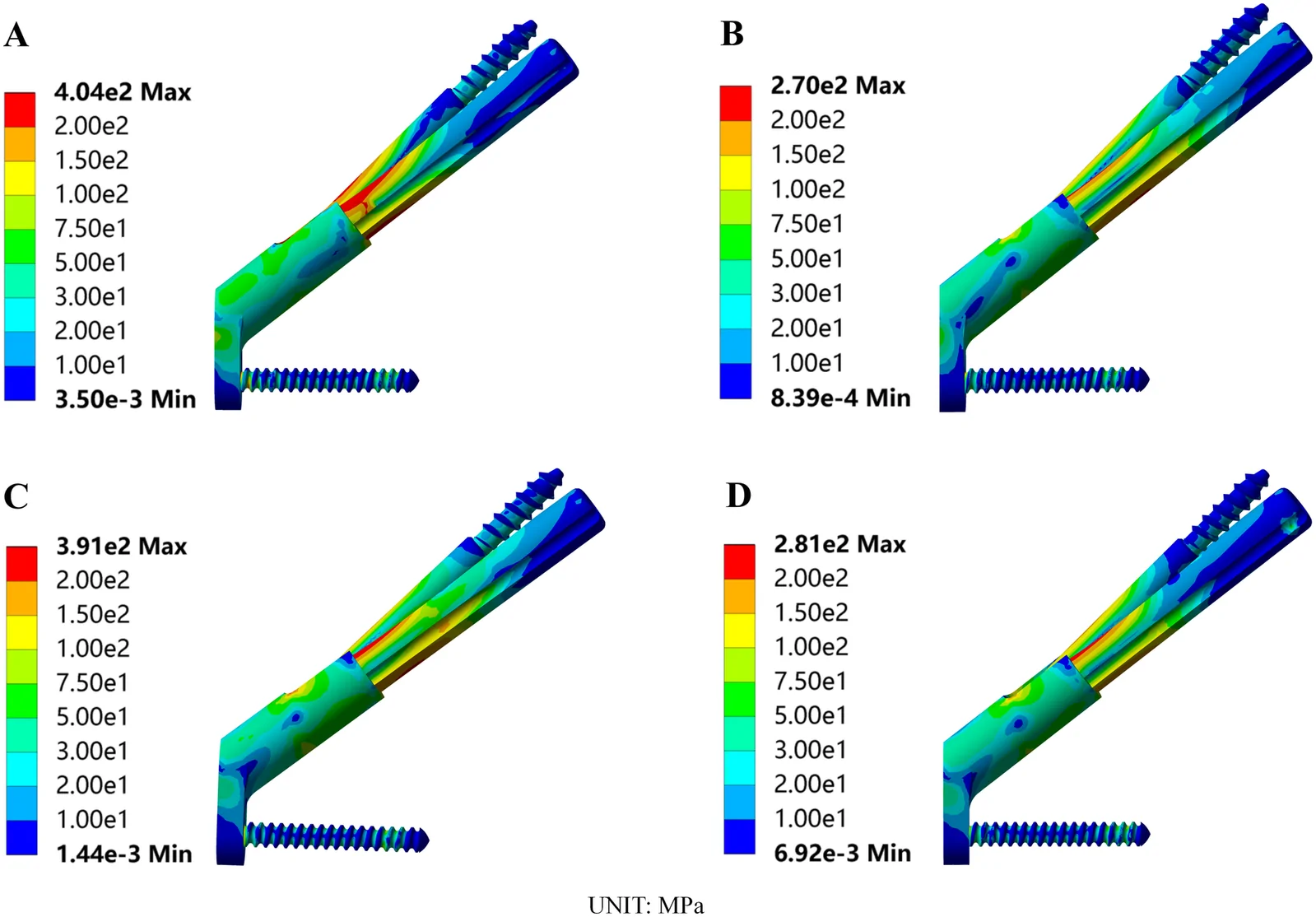
Stress nephogram of the FNS in the model with superior bone defect: **(A)** anterior bolt placement; **(B)** central bolt placement; **(C)** posterior bolt placement; **(D)** inferior bolt placement.

#### 3.1.3 Stress distribution on the femoral head

The peak VMS of the femoral head for the central positioning was 42.0 MPa. Anterior, posterior and inferior positioning increased this stress by 26.0%, 23.8% and 10.7%, respectively (Figure 5, Table 3). The central bolt placement strategy effectively relieved local stress concentration, thus lowering the risk of secondary fractures after surgery (30).

**Figure 5:**
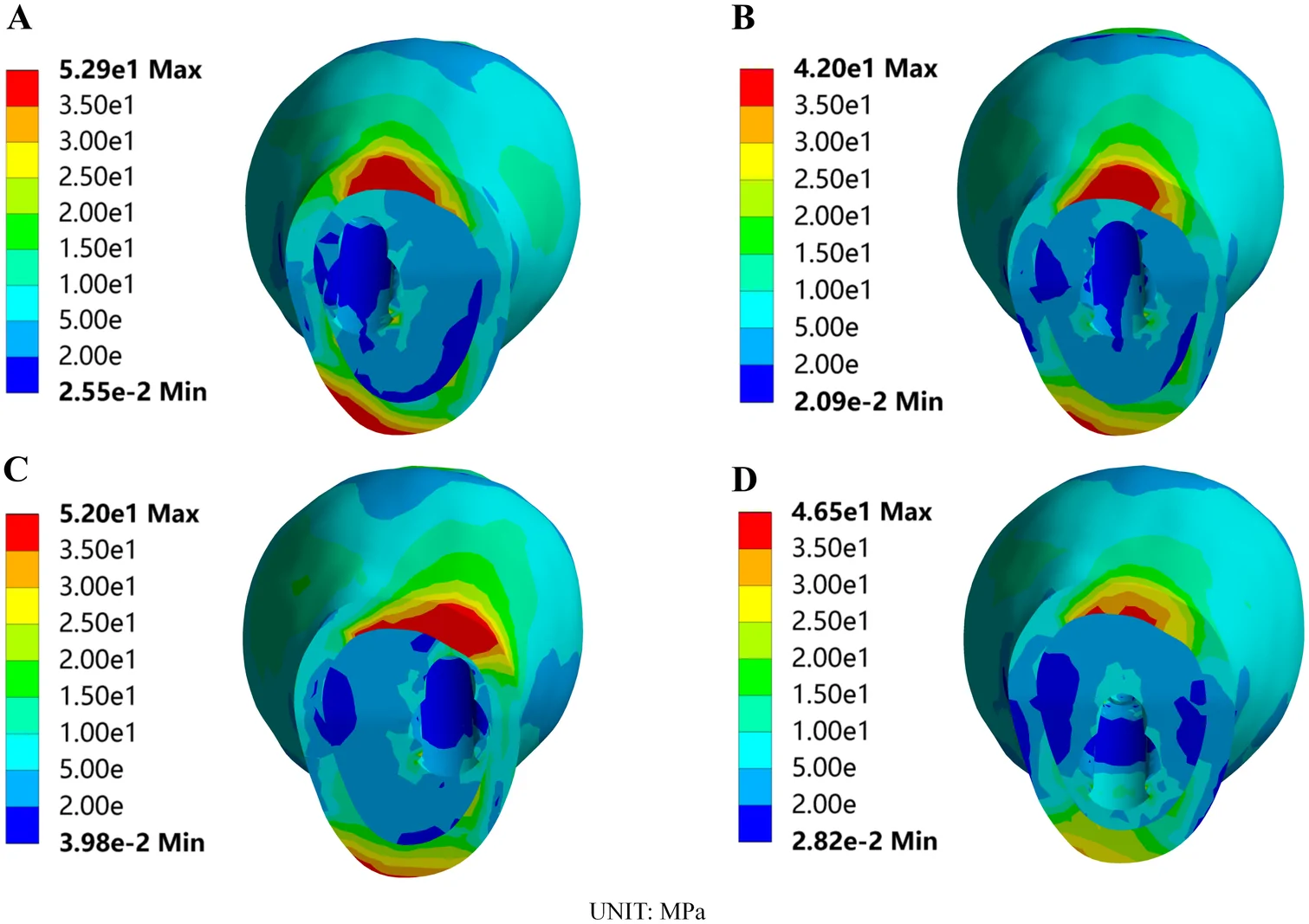
Stress nephogram of the femoral head in the model with superior bone defect: **(A)** anterior bolt placement; **(B)** central bolt placement; **(C)** posterior bolt placement; **(D)** inferior bolt placement.

#### 3.1.4 Interfragmentary relative sliding distance

The initial sliding distance for the central positioning was 0.289 mm. Anterior, posterior and inferior positioning significantly increased the sliding displacement by 84.4%, 48.4% and 3.81%, respectively (Figure 6, Table 3). Postoperative motion-induced displacement may reduce cortical support and compromise stability. Moreover, previous animal studies have shown that shear motion at the fracture site inhibits callus formation and delays healing (31, 32).

**Figure 6:**
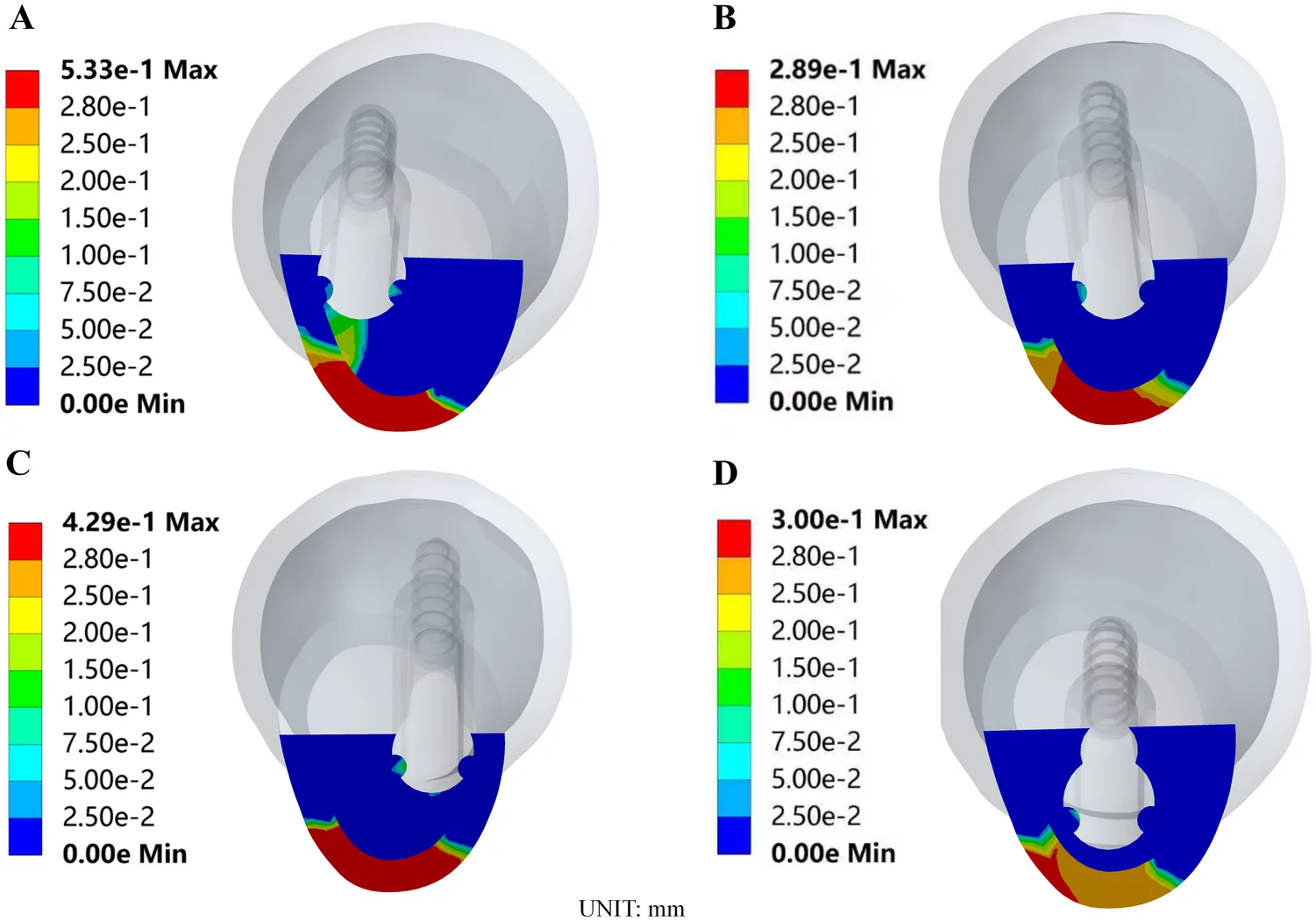
Relative sliding distance at the fracture ends in the model with superior bone defect: **(A)** anterior bolt placement; **(B)** central bolt placement; **(C)** posterior bolt placement; **(D)** inferior bolt placement.

### 3.2 Biomechanical results

Based on the experimental data, linear regression analysis was performed to characterize the relationship between displacement and load. The fitted linear models for the central and inferior FNS placement configurations yielded coefficients of determination (R^2^) of 0.97444 and 0.99357, respectively, indicating an excellent linear fit within the analyzed range.

Central FNS placement achieved an axial stiffness of 456.80 N/mm (Figure 7A), representing a 45.21% improvement over the inferior placement model (314.57 N/mm) (Figure 7B). In terms of ultimate load capacity, central placement reached 1420N, a 12.98% increase compared to inferior placement (1250N).

**Figure 7:**
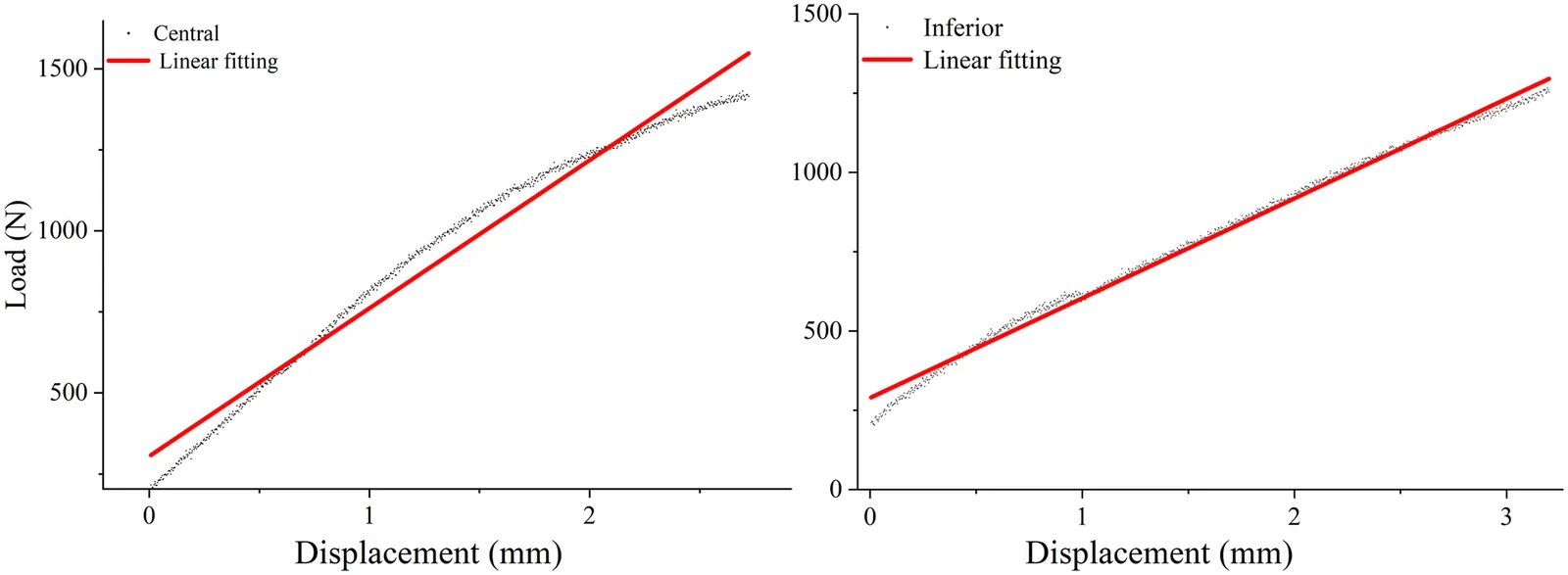
Linear regression of the load–displacement data obtained from biomechanical testing, with displacement (mm) plotted on the *x*-axis and load **(*N*)** on the *y*-axis. The slope of each fitted regression line represents the construct stiffness. **(A)** Central FNS placement: *y* = 456.80*x* + 304.77, corresponding to a stiffness of 456.80 ± 2.59 N/mm. **(B)** Inferior FNS placement: *y* = 314.57*x* + 288.97, corresponding to a stiffness of 314.57 ± 0.82 N/mm.

As shown in Figure 8A, the assembled cadaveric bone model was placed on the platform of the hydraulic testing machine in a physiological orientation. A centrally positioned FNS resulted only in limited collapsible fractures of the lateral wall, without catastrophic implant breakage or distal fractures (Figure 8B). Conversely, the inferior FNS placement strategy led to re-fracture at the distal locking screw site (Figure 8C). The mechanical results were consistent with the FEA findings: when the bone defect was located in the superior region of the femoral neck, the optimal FNS placement was at the center of the femoral neck.

**Figure 8:**
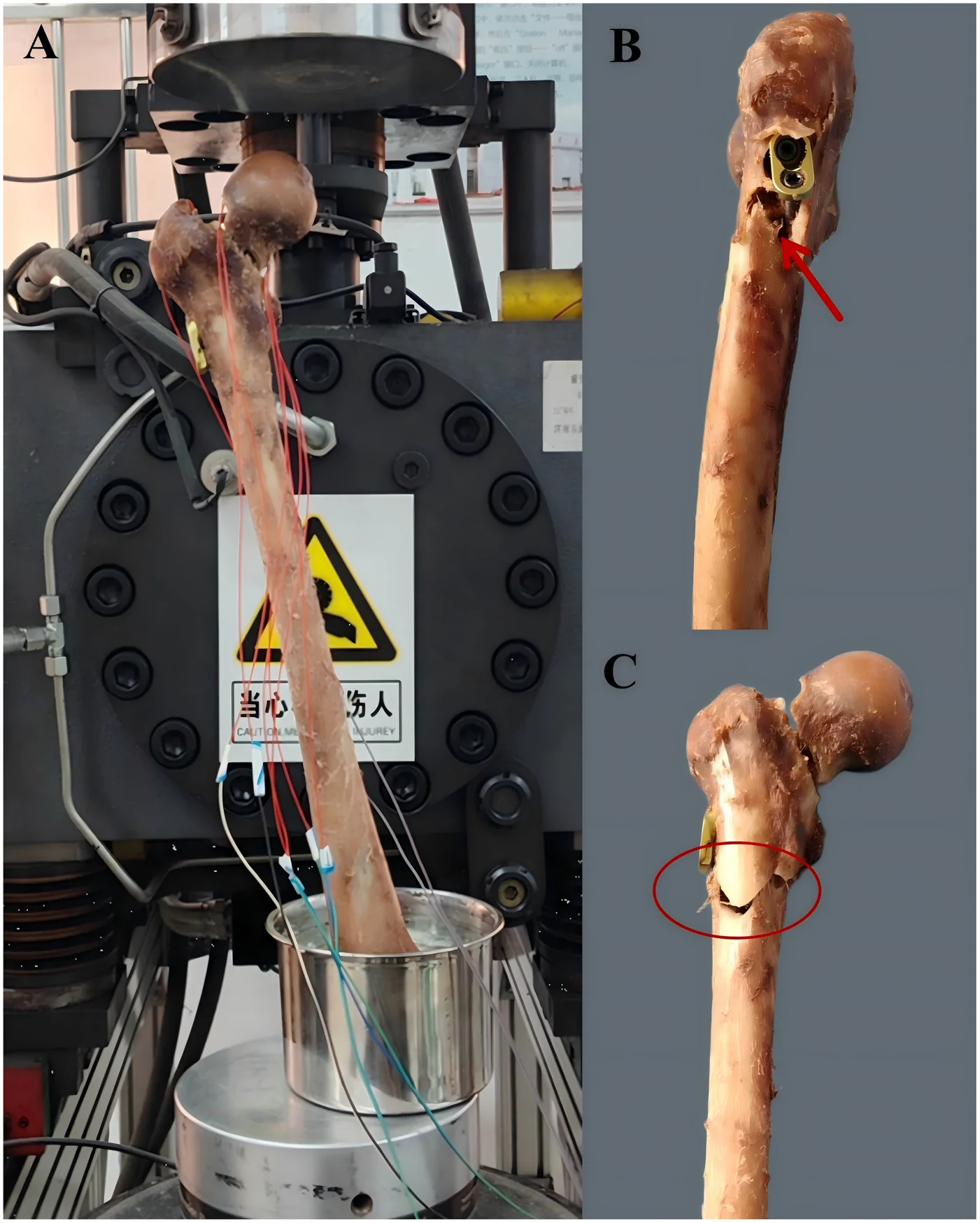
Biomechanical testing. **(A)** Experimental schematic diagrams of the biomechanical testing. **(B)** Lateral wall collapse in the central model. **(C)** Peri-implant fractures in the Inferior model.

### 3.3 Clinical outcomes

All patients completed the full 12-month postoperative follow-up. All patients with Pauwels type III femoral neck fractures complicated by superior neck bone defects underwent robot-assisted precise central implantation of FNS (Figures 9A–C). Immediate postoperative Garden Alignment Index assessment showed excellent reduction (Grade I/II) in all patients within this group (Figure 9D). CT scans clearly documented the fracture healing process in the superior defect group: continuous bridging callus across the fracture line was visible at 3 months postoperatively (Figure 9E). Besides, it is obvious that callus continues to form at six months, demonstrating excellent stability after fracture reduction (Figure 9F). Figures 9G,H show the anteroposterior and lateral x-ray views of the superior defect, demonstrating that the FNS internal fixation device was well maintained in its original position without cut-out at 12 months, exhibiting great robustness and reliable security. Radiographic follow-up at 12 months revealed complete fracture union and filling of the original bone defect, which was remodeled by newly formed bone tissue (Figure 9I). At the final follow-up, Harris Hip Scores in the superior defect group reached excellent/good levels, with no implant-related complications observed. Clinical results indicated no significant loss of neck-shaft angle or femoral neck height during the follow-up period.

**Figure 9:**
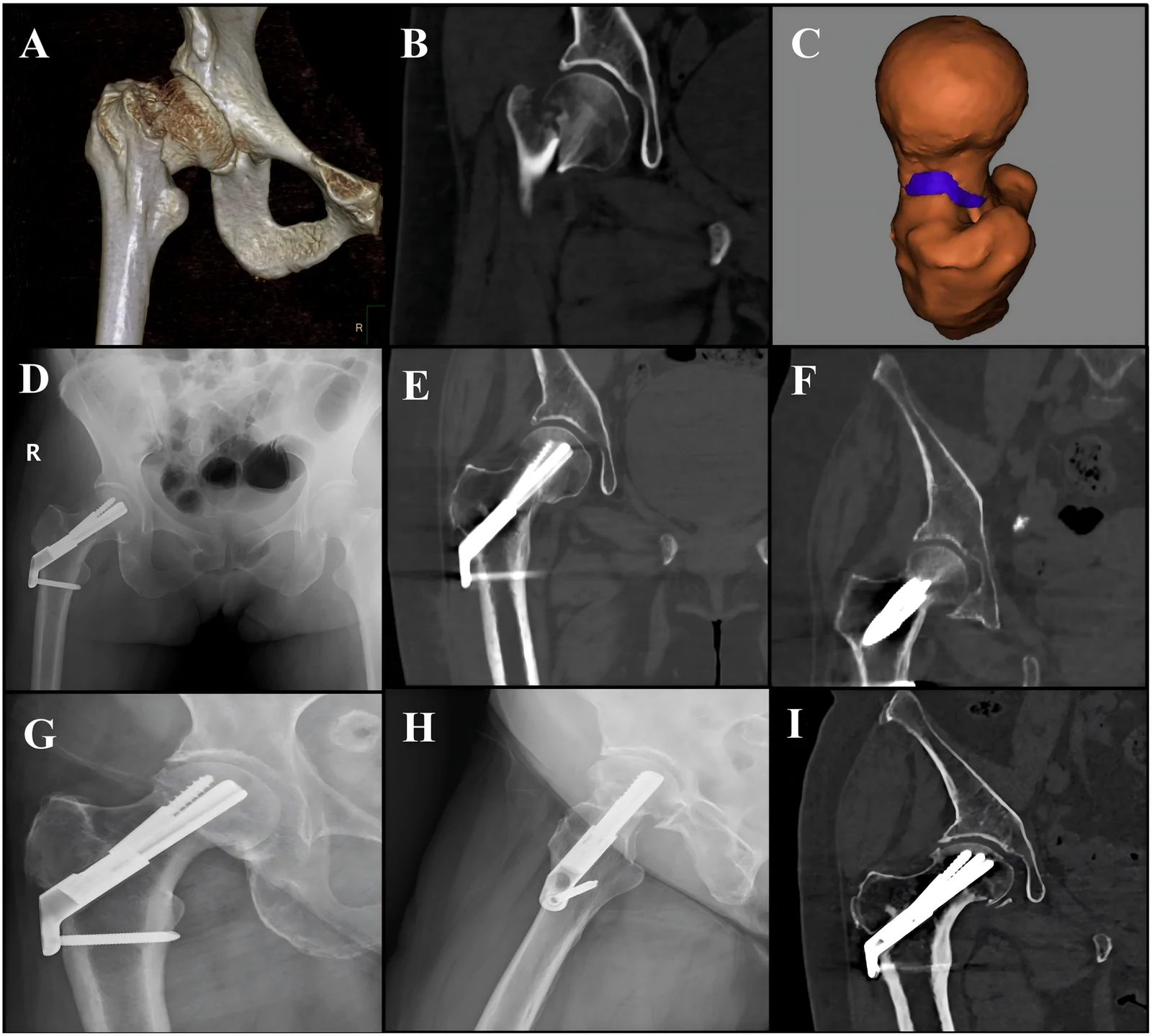
Imaging data of a patient with superior bone defect treated by central bolt placement. **(A)** Preoperative 3D reconstruction; **(B)** Preoperative CT scan; **(C)** Schematic diagram of the bone defect after virtual reconstruction; **(D)** Immediate postoperative bedside radiograph; **(E)** Three months postoperatively; **(F)** Six months postoperatively; **(G)** One-year postoperative anteroposterior radiograph; **(H)** One-year postoperative lateral radiograph; **(I)** One-year postoperative CT scan.

## 4 Discussion

Pauwels III femoral neck fractures with bone defects, resulting from high-energy injury, often inevitably increase fracture instability and face the difficulties of fixation, particularly in young and middle-aged adults (7, 10). From a biomechanical perspective, once a femoral neck fracture occurs, the osseous continuity of the superior aspect of the femoral neck is disrupted, signifying that the tensile side itself loses its physiological tensile load-bearing capacity. In a standard fracture without bone defect, successful anatomical reduction can re-establish bone-to-bone contact of the superior cortical fragments, enabling them to partially share compressive and bending loads during the stable post-reduction phase. However, when a bone defect exists in the superior femoral neck, this potential bone-to-bone buttress is completely lost, even if optimal alignment is achieved. Under the high vertical shear forces and bending moments characteristic of Pauwels III fractures, the lack of superior femoral neck bone support compels the implant to bear a greater share of the unmitigated extreme mechanical stresses, rendering the construct exquisitely sensitive to any surgical placement deviation. Consequently, the implant position must be chosen with extreme prudence.

First, FEA revealed that, either the anterior or posterior FNS placements exhibited poor biomechanical stability. Stress nephograms showed that these two placements induced pronounced stress concentrations in the FNS and the femoral neck cortex, which compromises the mechanical load transfer efficiency of the FNS and reduces post-reduction stability (30, 33). In addition, both models exhibited substantial displacement at the fracture site under axial loading. This abnormal stress distribution and instability indicate that excessive anterior or posterior placement of the FNS should be strictly avoided during surgery.

Drawing lessons from the above FEA results, the most suitable FNS position should compensate the biomechanical environment for corresponding bone defects. The central placement strategy provided maximum structural stiffness and ideal stress distribution for superior neck defects, whose peak stresses remained well below the material yield limit and thus reduced metal fatigue (22). On the other hand, in conventional surgical practice, positioning the femoral neck system (FNS) in a slightly inferior location within the femoral neck has been considered acceptable, as this helps to avoid excessive guidewire manipulation that may damage the bone. However, our results indicate that for fractures with a superior femoral neck defect, such inferior placement results in a severe mechanical error. When the FNS is displaced inferiorly, the stiffness of the overall construct decreases sharply by 45.21%. Cadaveric biomechanical testing further validated the actual stabilization effect of FEA by revealing the failure mechanisms associated with different FNS positions. In the superior neck defects, the centrally placed FNS only exhibited limited lateral wall collapse fractures, which did not lead to catastrophic implant breakage or distal fractures. Conversely, the inferior FNS position led to refracture at the distal locking screw. This severe complication can be attributed to a leverage-induced stress amplification mechanism. Because the superior neck defect leaves the tension side completely devoid of bony contact, the construct loses its force equilibrium, causing a inferior shift of the bending moment fulcrum. When the FNS implant is positioned too low, it behaves as an over-constrained short lever arm that absorbs the entirety of the uncontrolled varus thrust. Instead of being effectively dissipated through bone-on-bone contact, this substantial mechanical stress becomes markedly concentrated at the distal cortex perforated by the locking screw. Consequently, this interface experiences a critical stress concentration, ultimately exceeding the structural threshold of the cortical bone and triggering a secondary fracture. Conversely, strictly maintaining the FNS along the absolute central axis maximizes the working length of the implant and optimizes structural load dissipation.

Based on multimodal biomechanical and clinical findings, the central axis position represents an obligatory safety zone when treating Pauwels type III femoral neck fractures with superior neck bone defects using the FNS. Consequently, achieving precise central FNS placement remains highly challenging for orthopedic surgeons under limited visualization and inherent manual operation errors. To address this clinical difficulty, we employed the TiRobot system for high-precision trajectory planning and stereotactic execution, effectively eliminating the deviation associated with freehand FNS placement and ensuring strict adherence to the central axis. This integration of biomechanical investigation with robotic assistance provides a reliable and reproducible surgical approach for managing such complex injuries.

For Pauwels type III femoral neck fractures with superior bone defects, central FNS placement provides optimal structural stiffness and favorable stress distribution. Off-center placement, if performed without precise guidance, may induce pronounced local stress concentration and increase the risk of fracture fixation failure. Robot-assisted accurate central placement represents a reliable therapeutic strategy for managing this fracture pattern.

This study has certain limitations. First, the finite element modeling was simplified by omitting muscles and ligaments. Second, formalin-fixed cadaveric femurs were used instead of fresh-frozen specimens due to preservation constraints; moreover, the cadaveric biomechanical testing was limited by the small number of specimens and the lack of bone mineral density data. We therefore plan to increase the number of cadaveric specimens in future studies to further validate the biomechanical findings. Finally, because we isolated and focused exclusively on patients presenting with this specific superior defect morphology, the retrospective clinical sample size was limited to 27 cases. Future multi-center studies with larger cohorts are warranted to reinforce these clinical observations.

## Data Availability

The raw data supporting the conclusions of this article will be made available by the authors, without undue reservation.
